# Cerebrospinal Fluid Cavitation as a Mechanism of Blast-Induced Traumatic Brain Injury: A Review of Current Debates, Methods, and Findings

**DOI:** 10.3389/fneur.2021.626393

**Published:** 2021-03-12

**Authors:** Jenny L. Marsh, Sarah A. Bentil

**Affiliations:** The Bentil Group, Department of Mechanical Engineering, Iowa State University, Ames, IA, United States

**Keywords:** cavitation, BTBI, cerebrospinal fluid, blastinjury, injury-head trauma

## Abstract

Cavitation has gained popularity in recent years as a potential mechanism of blast-induced traumatic brain injury (bTBI). This review presents the most prominent debates on cavitation; how bubbles can form or exist within the cerebrospinal fluid (CSF) and brain vasculature, potential mechanisms of cellular, and tissue level damage following the collapse of bubbles in response to local pressure fluctuations, and a survey of experimental and computational models used to address cavitation research questions. Due to the broad and varied nature of cavitation research, this review attempts to provide a necessary synthesis of cavitation findings relevant to bTBI, and identifies key areas where additional work is required. Fundamental questions about the viability and likelihood of CSF cavitation during blast remain, despite a variety of research regarding potential injury pathways. Much of the existing literature on bTBI evaluates cavitation based off its *prima facie* plausibility, while more rigorous evaluation of its likelihood becomes increasingly necessary. This review assesses the validity of some of the common assumptions in cavitation research, as well as highlighting outstanding questions that are essential in future work.

## 1. Introduction

There have been over 383,000 traumatic brain injuries (TBIs) reported by military service members since 2000, with blast-induced traumatic brain injury (bTBI) being the most common injury (1). The bTBI occurs when a blast wave, created by detonating improvised explosive devices (IEDs), propagates through the head of an individual (2–4). Exposure to the blast wave can cause life-threatening injuries and fatalities, with the outcome depending on factors such as how close the individual is to the source and the overpressure (i.e., shock wave) amplitude created (3).

In the first three quarters of 2019 alone, 15,262 cases of bTBI were diagnosed, and these numbers only represent the first blast exposure reported by a given service member, meaning the number of injuries is even larger when repeated blast exposures to the same individual are taken into account (5). A single blast exposure, without secondary injury (e.g., blunt and penetrating injury, for example, due to shrapnel), can lead to pathological symptoms, cognitive deficits, behavioral changes, and mood disorders (6).

Despite an array of research on likely TBI mechanisms, there is not a clear and unified injury pathway in the case of blast injury (7, 8). Thus, hindering the critical design of optimal protective gear to mitigate bTBI and improve treatment plans for persons exposed to blast waves.

### 1.1. Review Scope and Rationale

One of the most commonly theorized bTBI mechanisms involves the formation and bursting of cavitation bubbles within the cerebrospinal fluid (CSF), appearing in literature as early as the 1950's (9). Cavitation refers to the formation and subsequent collapse of bubbles formed in response to local pressure fluctuations within a fluid. However, there is currently no conclusive evidence that realistic blast scenarios lead to CSF cavitation. Further, the specific manner through which cavitation may lead to cellular and tissue damage is similarly unclear.

Cavitation research has greatly expanded in the last 5 years, in terms of number of publications, as well as variety in study designs (10–13). Computational approaches and a range of experimental techniques are being applied to cavitation problems. These computational models and experimental outcomes are explored in later sections of this review, but range from finite element (FE) models to shock tube experiments. Research on cavitation as a bTBI mechanism has been conducted in a variety of fields, using multiple approaches and outcome measures. As such, the intention of this review is to collectively present the body of work regarding cerebrospinal fluid cavitation as a bTBI mechanism, while highlighting the relevant debates and unanswered questions. In doing so, this review will serve as a means to facilitate discussion among members in overlapping disciplines (e.g., biology and engineering) regarding CSF cavitation and its potential role as a bTBI mechanism.

Cavitation does not stand alone as the only posited explanation for the primary mechanism of bTBI. Other potential bTBI mechanisms include spalling, inertia, and implosion (14, 15). These alternate blast-body interactions (e.g., spalling, implosion, and inertial forces) may or may not occur concurrently with cavitation, as is discussed in section 3.4. The remainder of this section introduces the aforementioned blast-body interactions and provides statements for why CSF cavitation was selected as the focus of this review.

Spallation may occur when a shock wave travels from a more to less dense media, which may cause fragmentation of the denser material into the less dense one (16). Literature of experiments performed to investigate spalling within the brain is limited. Rather, existing literature of spalling in biological systems primarily focuses in air containing organs like the lung and bowel (17, 18). Further, more recent work suggests that spalling may not be as likely for a blast-brain interaction when compared with gas containing organ systems. The reason being because the brain's solid-fluid interfaces demarcate comparatively smaller density changes (19).

Theoretical understanding of implosion is enhanced by cavitation research since the two phenomenon are related (16). As such, the cavitation bubble collapse-related findings discussed later in this review can be cross-applied to the discussion of implosion. However, like spalling, the literature shows that implosion is more likely to occur in the gas containing organ systems (18). Implosion is inherently discussed in section 2.2 since collapse of existing nanobubbles within the brain may be considered as implosion, rather than cavitation. However, the existence of nanobubbles within the brain, and their implosion, are not directly or indirectly experimentally confirmed.

Inertial forces at the interfaces between medias of different densities may also cause bTBI. Specifically, shearing injury may occur when the blast wave accelerates the medias at different rates (16). Injury due to inertial forces is relevant in traumatic brain injury research, but is not the focus of this review since research regarding inertial forces in the context of blast injury is limited to the extent that a review would not be beneficial (20).

It must be noted that there is no conclusive experimental evidence at this time linking potential blast-body interactions (e.g., spalling, implosion, and inertial forces) to bTBI. However, there is experimental and computational data supportive of cavitation effects (16). Additionally, cavitation has the largest body of literature when compared with other bTBI mechanisms such as spalling, implosion, and inertial forces. Thus, cavitation serves as a starting point for an interdisciplinary review of techniques and findings in the context of bTBI.

**Figure 1:**
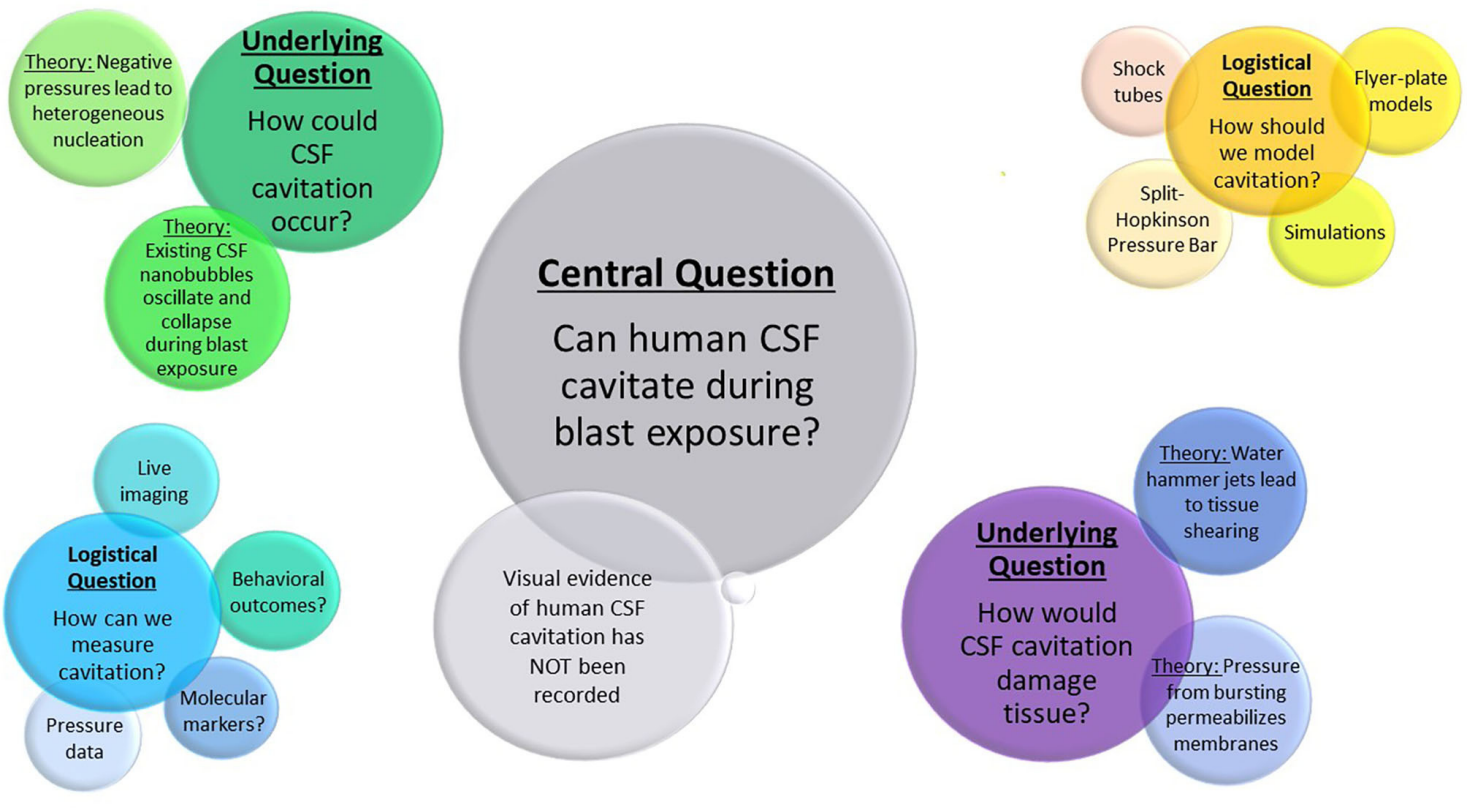
Outstanding questions and debates in cavitation research described in this review. The largest bubble represents the central question of cavitation in bTBI research. Underlying questions and their theoretical solutions are labeled in the second largest bubble clusters, with logistical questions and possible approaches shown in the smallest clusters.

Section 2 addresses whether cavitation could occur within the CSF, and potential ways this could occur based on other biological examples of cavitation. Section 3 discusses possible injury mechanisms through which cavitation could lead to cell and tissue damage, as well as other potential mechanisms which may or may not occur in conjunction with cavitation. Section 4 highlights experiments and simulations used to model cavitation in research. The approaches and outcome measures used to assess cavitation damage are presented in Section 5. Finally, Section 6 provides the concluding remarks and future directions, addressing the critical questions remaining to be answered, as well as considerations for new work in the field. The key debates and questions examined by this review can be found in Figure 1.

## 2. How Do Cavitation Bubbles Originate in the Cerebrospinal Fluid?

The cerebrospinal fluid system contains approximately 140–150 mL of CSF, with measurements usually centering around 50 mL in the lateral ventricles (21). As such, the ventricles represent the largest region filled with CSF in which cavitation could occur. However, were cavitation to occur exclusively within the ventricles, it would be difficult to explain cavitation-related tissue damage outside of the ependymal cells that make up the ventricle's epithelial lining. Furthermore, although less likely and certainly not impossible, is cavitation causing damage in the vasculature. The volume of the CSF far exceeds the volume of blood inside the vasculature, in any given vessel, and the blood flows much faster than CSF circulates (22). There is also a difference in the ionic composition of blood compared with CSF. Differences in volume, geometry, flow rate, and ionic composition mean that blood could have distinctive cavitation-related damage characteristics when compared with CSF. For instance, cavitation within the vasculature is unlikely to lead to tissue shearing or parenchymal lesions one might associate with bTBI (23, 24). Rather, one would expect to see weakening or even leaking from the vessels. This could be consistent with some of the findings discussed in section 4, which mention that cavitation damage appears to propagate along the vasculature. However, research on cavitation within the vasculature, in the context of bTBI, is extremely limited in comparison to the study of CSF cavitation. Hence, cavitation in the vasculature takes on a minor role in this review.

Cavitation can also occur within soft materials and also in the solid portion or within the liquid portion of a tissue (25–28). In fact, cavitation within the brain matter may be a better logical explanation of bTBI. This is because injury attributed to shearing throughout the brain would be more consistent with current damage models (29–31). Thus, the bTBI would not be limited to surfaces in contact with the CSF layer. However, cavitation rheology (32) and research on cavitation in biological tissues (33) are newer and less well-documented nuances. As a result, this review will focus on CSF cavitation due to the paucity of literature on cavitation in the brain (31, 34–36). Additionally, the CSF is the focus of this review since the CSF is the largest and ionically simplest fluid in the brain. Thus, making the CSF a plausible site of widespread cavitation. However, there are many unanswered questions about CSF cavitation, as highlighted in this review. These unanswered questions imply that large advancements need to be made in developing approaches for researching cavitation in biological fluids, before more nuanced cavitation in other areas of the brain and vasculature can be adequately addressed.

The next two subsections discuss the prevailing theories on how cavitation could occur within the cerebrospinal fluid. The first such theory is that bubble nucleation is enabled by the conditions generated by the blast wave propagation through the skull. The second is that blast exposure leads to oscillation and eventual collapse of existing nanobubbles within the CSF. The remaining subheading discusses these possibilities in light of other known instances of biological cavitation.

### 2.1. Theory: Blast Wave Exposure Leads to Cavitation Bubble Nucleation

**Figure 2:**
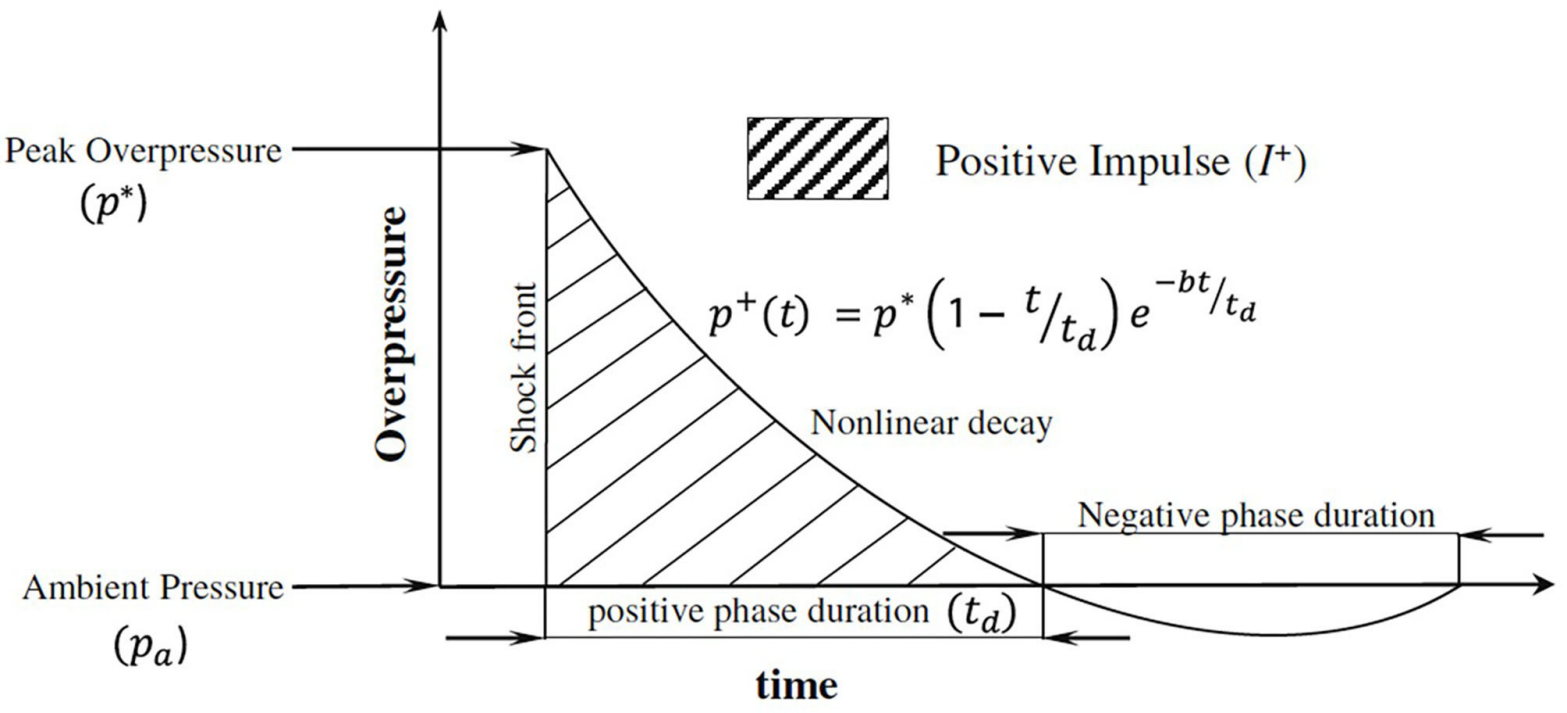
Planar Friedlander waveform adapted from Chandra et al. (37). In the figure, the instantaneous overpressure *p*^+^ at time *t* is defined using the peak overpressure p^*^, positive phase duration *t*_*d*_, and decay constant *b*.

Shock wave experiments and simulations typically model a free-field explosion, where the blast wave is described using the Friedlander waveform (Figure 2). The Friedlander wave has a positive pressure phase that includes a shock front, which is followed by nonlinear decay containing a negative pressure phase before returning to ambient conditions (37).

Negative pressure, or a drop below local vapor pressure, is the necessary condition for cavitation (38). Therefore, the potential to cause cavitation exists since there is a negative pressure component of an incident blast wave modeled using the Friedlander waveform. As the negative portion of the blast wave travels through the skull, the pressure within the CSF may drop below the cavitation threshold and form bubbles (34, 39). This CSF cavitation could theoretically be an instance of homogeneous nucleation, but is more likely heterogeneous (i.e., nucleation preferentially occurs at certain sites) (40). After the negative portion of the blast wave travels through the skull, restoration to local pressure causes bubble bursting (29, 41).

Skull flexure is another condition that may influence local pressures and enable nucleation (42, 43). Flexure can occur when a stress (i.e., pressure) wave travels through the skull faster than the blast wave, which may result in sufficient negative pressure at the contrecoup site leading to nucleation. The propagating stress waves can also generate flexural ripples of the skull (44). Using a surrogate head model in a shock tube environment, Ganpule et al. (43) found that the skull shell deformed up to 0.18 mm over the course of 2.5 ms when exposed to a blast wave. The peak surface overpressure, measured by the sensor placed on the forehead, was 0.55 MPa. Salzar et al. (42) utilized *post-mortem* human heads and a shock tube setup to cause flexure (or deformation) at the front of the skull when subjected to a blast wave with a 140 kPa overpressure and a pulse duration 2–4 ms. This blast wave also caused cavitation in a CSF simulant (i.e., saline) at the contrecoup site of the head, as evidenced by the negative and positive pressure time traces measured by a pressure transducer that was placed at the countercoup location inside the skull (42).

As the pressure wave travels through the head (e.g., skull–CSF–brain system), reflections at the boundaries (e.g., skull–CSF or CSF–brain) of acoustic impedances may occur. These reflections are due to the different material properties of the skull, CSF, and brain. For instance, a positive pressure wave can be converted to a negative pressure wave due to the fixed end reflection at the skull–CSF boundary. This conversion could result in a negative reflection sufficient for nucleation.

Whether cavitation is created due to the incident blast wave, skull flexure, and/or reflection from the boundaries of acoustic impedances, the pressure distribution in the head will be influenced and will affect cavitation formation. The bursting of cavitation bubbles could lead to tissue damage via several possible mechanisms, described in section 3. The specific cavitation threshold of cerebrospinal fluid, and its potential to cavitate at non-lethal blast pressures will be discussed more in section 4.

### 2.2. Alternative Theory: Pre-existing Nanobubble Oscillation

In contrast to the theory that blast conditions enable nucleation, Adhikari et al. (10) has suggested that nanobubbles naturally occur and may already exist within the brain. In such a case, propagation of the blast wave through the skull could lead to oscillation and collapse of these existing bubbles. Thus, an alternate explanation for bTBI attributed to cavitation is the oscillation and bursting of these nanobubbles. Specifically, propagation of the shock wave through the skull would lead to oscillation and bubble collapse (45). This possibility could enable cavitation bursting at blasts with negative pressure magnitudes (e.g., 50–200 kPa) that might otherwise be too insufficient to enable nucleation (46).

### 2.3. Lessons From Other Instances of Biological Cavitation

The characteristics of cavitation are a function of the source causing the bubble nucleation. This subsection provides examples of sources (e.g., shock wave lithotripsy, extracorporeal shock waves, and ultrasonic cavitation) used to generate cavitation in biological or medical settings. The cavitation characteristic from these sources are different from those created by a real blast wave. For instance, a typical positive pressure duration of extracorporeal shock waves is on the order of 1 μs (47), while that of real blast shock wave is in the range of 1–10 ms (48). Additionally, ultrasound waves may not resemble the Friedlander wave (Figure 2), but rather have waveforms that are periodic with limited bandwidth (49). Ultrasonic shock wave applications also generally have a very small focal target, as opposed to a blast injury where the whole body is exposed to the shock wave. An example of a typical acoustic shock wave for medical application highlights the differences between these therapeutic techniques and a real blast wave. For instance, an acoustic shock wave has a range of −8 to 40 MPa over 1.5 mm, where the positive pressure lasts 1 μs (50). Given these highly divergent conditions, the applicability of ultrasonic cavitation research to blast injury should be considered conservatively. One argument for utilizing ultrasonic or commercial extracorporeal shock models, is to examine microscopic level injury that a more realistic blast model is not well-suited to produce (51). However, the results from ultrasonic microscopic models may not be generalizable to blast injury, due to the different focal areas of the shock wave exposure, overpressures, and pressure durations.

Shock wave lithotripsy provides some support for the idea that microbubbles may already exist within the body. For example, intestinal hemorrhage has been observed after oscillation of existing gas bodies, without injection of any microbubbles. This could be suggestive that there are analogous gas bodies within the CSF, which may burst upon oscillation (52). Medical applications like lithotripsy generally have higher pressure-amplitude shock waves, with average frequencies of 200 kHz whereas diagnostic imaging apply frequencies ranging from 1 to 20 MHz (52). Studies using lithotripter shock waves in mice found that the threshold for intestinal hemorrhage in mice was 100 shock waves at 1.6–4.0 MPa, with a center frequency of 2 MHz (52).

Extracorporeal shock waves (ESW), similar to those used for lithotripsy, have also been used in more than one instance to generate bTBI due to cavitation (31, 53). In one of those instances, bubbles were artificially injected to increase cavitation effects, suggesting likely bTBI due to bubble collapse (31). Each animal was injected with 0.02 mL/100 g of ultrasound contrast (microbubbles) and were then exposed to a range of ESW conditions after approximately 20 s. The shock wave conditions (1 Hz) varied in number of shock wave exposures (1, 2, 4, 8, 12) as well as intensities ranging from −7 to −14 MPa peak negative pressures (31). These conditions led to blast-representative parenchymal lesions, but did not mimic a real blast in duration or pressure. Experiments by Divani et al. (53) used 5 or 15 shock wave exposures, also at 1 Hz, with peak negative pressures of −10 MPa. These ESW did lead to graded cognitive symptoms in the animals, but the repeated, short duration, high pressure shock waves may not lead to the same types of diffuse neuronal injury expected from a blast wave (53). In studies by both (53) and (31), shock waves were able to generate characteristic bTBI histopathologies, like tissue shearing and parenchymal lesions. Although, it is unclear how the magnitude of the bTBI histopathologies would be different if a real blast wave was used to induce cavitation. Thus, making correlation a challenge without experiments of blast-wave induced cavitation.

Use of ultrasonic cavitation in the disruption of amyloid fibrils has also demonstrated that hydrophilic residues in trans-membrane regions can act as bubble nucleation sites, supporting the possibility of heterogeneous nucleation during blast exposure (54). The molecular dynamics (MD) simulations by Okumura and Itoh (54) showed that the experimental standard of 0.1 MPa at 20 kHz were lower than the parameters needed to disrupt the fibrils. In the MD simulations, bubbles formed at pressures greater than −200 MPa and burst at positive pressures up to 400 MPa.

Inertial cavitation, which occurs when a bubble collapses violently and produces a shock wave, is not a necessary condition to observe effects like blood brain barrier (BBB) disruption. BBB disruption can be achieved at 260 kHz (repetition frequency 1 Hz), with peak pressures from 0.1 to 0.6 MPa and burst lengths of 20 ms over a 20 s trial (55). However, this does not mean cavitation is not occurring in general, and the presence or absence of inertial cavitation is independent of the other cavitation mechanisms (e.g., acoustic cavitation or nanobubble implosion) discussed in this review (55).

## 3. Potential Mechanistic Pathways of Cavitation in bTBI

Even if the preceding literature is taken as sufficient, it remains unclear how cavitation bubble formation and/or collapse leads to cell or tissue damage. The prevailing theories are described in the following subsections.

### 3.1. Water Jets

One of the most prevalent proposed cavitation mechanisms is the formation of water jets or water hammer jets upon bubble collapse (50, 56). Water jets can lead to tissue shearing and tearing consistent with bTBI, which may be unique to this form of injury (24, 57). Water jets have also been shown to disrupt tight junctions and lead to changes in cell morphology (58). It has been experimentally demonstrated that artificially placed bubbles on rat hippocampal slices did in fact produce water jets upon bursting (12).

### 3.2. Damage From Bubble Collapse

An alternative damage mechanism, which could occur independently from or in conjunction with water jets, is the damage generated from the collapse of the bubble itself. Upon collapse, cavitation bubbles can also create shock waves (i.e., secondary shock waves) which generate overpressures that may be even greater in magnitude than the original (i.e., primary) blast wave that caused cavitation (23, 34). For instance, a simulation by Haniff and Taylor (35) showed that the local secondary shock wave overpressure was five times greater compared with the primary blast wave overpressure of 400 kPa. The secondary shock waves could result in cell membrane damage, discussed below, as well as parenchymal lesions. Bubbles also generate heat upon collapse, which could lead to direct damage or inflammatory response (59, 60).

### 3.3. Cell Membrane Sonoporation

Whether as a result of water jet formation or direct damage from bursting, cavitation has been conclusively shown to permeabilize cell membranes (12). In particular, cells subjected to shock tube experiments have been compromised to the point that macromolecules can be delivered into cells (61, 62).

Molecular dynamics simulations of nanobubble collapse near a lipid bilayer showed that bursting leads to invagination and increased water permeability (63). Cavitation collapse has also been shown to lead to ion channel dysfunction and ionic imbalance even in the absence of membrane poration (64).

### 3.4. Cavitation-Coupled Mechanisms

There are, of course, other theories on how bTBI damage occurs. However, it is worth noting that many of these theories could exist in conjunction with, or perhaps even cause, cavitation. Rapid transmission of blast pressure through the vasculature (thoracic surge) could lead to disruption of vessels including those in the blood-brain-barrier (30, 57), with pressure changes within the vessels leading to cavitation. This theory could explain the finding that tissue shearing in cavitation simulations propagates along the vasculature (65). In addition to thoracic surge, blast effects (e.g., skull flexure, spalling, implosion, and inertial forces that were discussed in section 1.1) could occur in conjunction with CSF cavitation. Cavitation could even theoretically be occurring as a secondary mechanism caused by these other effects, rather than in response to primary blast exposure itself. Furthermore, deformation of the brain that is attributed to skull flexure or direct transmission (66, 67) could still enable or even exacerbate negative pressures that might surpass CSF's cavitation thresholds. Additionally, cavitation may also be a unifying element of several theories on injury mechanisms, since deformation and pressure changes in many of the theories could lead to bubble collapse.

## 4. Methods for Studying Cavitation

The following subsections address common experimental and computational approaches being used to study cavitation, some relevant findings, and potential benefits and challenges for the model choices. It is worth noting that simulating blasts experimentally or computationally can prove difficult, due to a lack of accessible information detailing positioning and force of blasts that occur in combat (68). Since the pressure limits of bTBI are not completely determined, the planar shock wave set up for many shock tube experiments may not accurately reflect complex blast scenarios.

In some of the experiments and simulations discussed below, the pressures used far exceed what would be considered a lethal blast pressure. Typically, 690 to 1,720 kPa peak overpressure is considered potentially lethal (14). Thus, experiments and computational models that are performed using pressures that exceed 690 kPa are not informative for understanding bTBI mechanisms due to cavitation since these pressures would yield a fatality. As a result, future experimental and computational models should consider pressures that are survivable, when investigating bTBI scenarios that may generate cavitation bubbles.

Only one of the experiments described below actually use or model the parameters of cerebrospinal fluid obtained from porcine. The rest of the experiments mentioned use water or physiological saline as a simulant for CSF. The choice of model for CSF (real vs. simulant) is important because the cavitation threshold of CSF has not been experimentally determined. In fact, the only study of cavitation using cerebrospinal fluid that was conducted indicates that porcine CSF has a divergent cavitation threshold from water (11). Thus, generating cavitation bubbles in water due to non-lethal overpressures would require a negative pressure that does not surpass the cavitation threshold of water (69, 70). These critical modeling issues (e.g., choice of overpressure and negative pressure magnitudes, selection of CSF or CSF simulant), as well as the varied research approaches described below, make it clear that there is no standard approach for biological cavitation research. Therefore, highlighting the need for realistic modeling of blast scenarios and CSF properties.

### 4.1. Experimental Methods

There are several common experimental approaches used to model blast or blunt impact, to investigate cavitation scenarios. A summary of experimental works described in the following subsections can be found in Table 1.

**Table 1:** A summary of experimental studies conducted on brain injury using cavitation.

**Experimental method**	**Sample studied**	**Outcome measure**	**Duration given**	**References**
Blunt impact	Acrylic resin, water and agar head model, collision and strike set ups for impact	Pressure data, up to 400 kPa change observed	Pressure duration not given, cavitation recorded over 3 ms	(71)
Extracorporeal shock wave (ESW) device	PiezoWave ESW device utilized to apply shock wave (48.1 MPa) to anesthetized Spague-Dawley rats	Cellular assays (viability, division, signaling)	Pressure duration not given	(31)
FlyerPlate	Cell (neuroblstoma, glioma, epithelial) monolayers are impacted by copper fragments to generate shock wave trauma	Cellular assays and imaging- mitotic activity, cell death	Pressure duration not given, cavitation recorded from 5 to 500 μs	(13)
Shock tube	Polycarbonate, water, Sylgard gel head model, 18-in. shock tube	Strain data, overpressure range 69–170 kPa	Pulse duration 2–4 ms	(34)
	Polycarbonate and water head model, 28-in. shock tube	Pressures/strains, peak overpressure 0.13 MPa	Positive duration 4.55 ms	(72)
	*Post-mortem* human subject heads filled with saline/ballistic gel, 18-in. shock tube	Pressure, high speed images, overpressures 100–350 kPa	Positive duration 5 ms	(42)
	Gel filled ellipsoid head surrogate, 12-in. shock tube	Pressure and strain data	Duration not given	(39)
Split Hopkinson pressure bar	Compressive modified cavitation Hopkinson bar, water and porcine cerebrospinal fluid with seed bubbles	Strain and velocity data, up to 3.5 MPa fluid pressure	Negative pressure duration 0.15 ms	(73)
	Customized SHPB used to oscillate a single seed bubble placed in *ex vivo* hippocampal slices	Pressure and histological data, Digital Image Correlation	Overpressure duration ~540 μs	(12)
	SHPB, acrylic chabmber filled with water and hydrogel slices with seed bubbles	Imaging data, DIC	Positive pressure duration 550–750 μs	(23)
	Hydrogels and rat brain slices with seed bubbles [similar to (12)]	Pressure, Image data, DIC	Pressure duration not given, bubbles recorded from 270 to 350 μs	(74)

#### 4.1.1. Blunt Impact Models and Flyer Plate

Blunt impact models can generate cavitation, and as such are still sometimes utilized even when studying brain injury (71). For example, an acrylic resin container filled with agar and water to represent the skull, brain, and CSF, respectively, was rapidly accelerated into a wooden wall in one experiment and impacted with a moving striker in a different experiment (71). Collision speeds of 5 and 10 km/h generated cavitation damage, even in the absence of container (skull) flexure, or penetrating injury (71). These blunt impact models had negative pressures that appeared 2.5–3 ms after collision, with a peak positive pressure of 400 kPa.

Controlled cortical impact (CCI) is another common method for modeling brain injury, and is most often conducted by accelerating an impactor into the exposed cortical dura at various velocities ranging from 2 to 6 m/s (75). The severity of damage is controlled by varying the sizes of the impactor, which can have a diameter of 3 to 15 mm. The size of the impactor depends on the animal choice, which can range from rats to non-human primates (75). However, controlled cortical impact is less applicable in shock scenarios because the craniotomy usually conducted to expose the dura is unrealistic for a blast injury, and the process is traumatic in itself (76). On the other hand, indirect impact to the brain (e.g., head models which include the skull, or fluid surroundings to simulate CSF) can produce shock waves within the tissue that more realistically model a blast scenario without introducing additional trauma to the tissue as in the controlled cortical impact approach. As such, a more appropriate shock-induced cavitation model may be the flyer plate. With the flyer plate, a laser-driven copper fragment can impact cell culture dishes; thus, generating a shock wave within the media (13). In the study by Cao et al. (13), the laser (1,064 nm) produced bursts of energy at 500–600 mJ. Cavitation was observed from 5 to 500 μs, following excitation. The maximum mean peak pressure of the vial containing cells and media was >70 MPa.

Blunt impact models have been used to demonstrate cavitation. However, blunt impact cavitation experiments are still not particularly valuable for studying blast injury due to the divergent nature of the loading mechanism. For example, the flyer plate model provides a means of analyzing the effects of cavitation on cells. Yet, the flyer plate does not result in realistic blast pressure profiles. As such, these models are not generally ideal for studying biological cavitation, or blast injury.

#### 4.1.2. Split-Hopkinson Pressure Bar

A Split-Hopkinson Pressure Bar (SHPB) has been used to create cavitation in experimental settings (12, 73, 74). This experimental set up typically involves a striker bar that impacts an incident bar to create a pressure wave that is traveling at 2,230 m/s (77). The pressure wave typically has a magnitude of 1 MPa and a duration of 2–5 ms (12, 73, 74). This pressure wave is transmitted to a fluid-filled test chamber with dimensions of 90 × 50 × 40 mm (12). The fluid in the chamber could be water, human CSF, or porcine CSF.

Utilizing the SHPB method, it has been shown that bubble collapse leads to much higher strains on a tissue-like hydrogel than strains attributed to the passage of the initial shock wave (23). Strains for the tissue-like hydrogel due to bubble collapse are on the order of 0.21–0.51, compared with <0.002 for the incident shock wave. The SHPB method has also been used to burst artificially placed bubbles on rat hippocampal slices, where bubble collapse led to pathological changes and diffuse focal injury (12). The diameter of the bubble was <0.2 mm, while the dimensions of the hippocampal slices were <11 × 9 × 1 mm. In the SHPB studies, a single seed bubble placed on the tissue slice oscillated and collapsed at 1 MPa overpressure and −0.2 MPa underpressure. The studies by Canchi et al. (12) attempted to be more biologically accurate, by using artificial CSF (aCSF) and a tissue slice in a pressurized chamber.

Tissue injuries have also been demonstrated in the rat brain, with characteristic tearing seen even as a result of the collapse of a single seed bubble, using the SHPB method (74). A SHPB is also utilized by Bustamante and Cronin (73), to visually confirm formation of cavitation bubbles in porcine CSF. Bustamante et al. (73) found a 50% cavitation threshold of −0.47 MPa for porcine CSF, which is indeed divergent from the literature estimate of −1.37 MPa for distilled water (73). Based on these results, a peak overpressure of 660 kPa would yield slightly above a 50% chance of cavitation in degassed cerebrospinal fluid, with an 89% chance in non-degassed CSF. Although the comparability of porcine and human CSF must be further characterized, this data would be suggestive that cavitation could occur at non-lethal blast pressures. However, the results of this model pertain to the case when the subject is substantially closer to the source force than a human would be to the source of an explosion. Additionally, the model considers only the fluid layer and not the influence of the skull and tissue on cavitation bubble formation.

Overall, the SHPB model provides some benefits over CCI and flyer plate models since realistic blast overpressures can be achieved. One important step in future research will be to combine some of the approaches mentioned in the above papers, to gain a complete picture of cavitation. For example, in some instances, the model is more biologically representative through the use of tissue slices, aCSF, and pressurized chambers. However, the use of a seed bubble makes extrapolation of the results for interpretation of cavitation bubble nucleation in a realistic blast wave scenario challenging. In other studies, CSF is able to cavitate, but the model lacks biological realism. In either case, the overall biological complexity is an issue that needs addressing, since none of the existing models in the literature realistically encompass the head geometry and material properties.

#### 4.1.3. Shock Tubes

Shock tubes may be a logical choice to model blast waves, but complications can arise due to the generally limited number of facilities that possess space and safety measures for the shock tube, as well as the biosafety precautions needed to test biological samples (68). However, such models can be very useful for the additional information they provide by simulating the interaction of the propagating shock wave with the head, to reproduce bTBI.

Using a shock tube and a simplified polycarbonate head model, Hua et al. (72) determined that anterior pressure was predominantly determined by blast wave direction, but pressure at the posterior of the skull was additionally affected by flexure. Pressure at the anterior and posterior of the skull was 0.25–0.3 MPa and 0.0–0.02 MPa, respectively. The cross-sectional area of the shock tube used by Hua et al. (72) was 711 mm, with a length of 12,319 mm. The shock wave, created by bursting the Mylar diaphragm, had an overpressure of 0.13 MPa and a positive duration of 4.55 ms. The polycarbonate head model had an inner diameter of 152.4 mm and thickness of 1.27 mm.

Shock tube experiments, involving full size head models or even *post-mortem* human heads, are essential for validating computational models predicting the response of the head to a blast wave (39, 42). The shock tube is a practical method for gathering pressure data to inform simulations and models of cavitation onset (34). Additionally, shock tubes provide the best scaling options for modeling realistic blasts, and are large enough to accommodate full head models that are biofidelic such that the response to a blast can be accurately analyzed. Furthermore, the shock tube facilitates examination of criteria that may influence the severity of a blast injury, such as distance from the source, duration of the blast, peak overpressure, reflections, etc. For cellular level applications, or for analysis of a fluid's cavitation properties, flyer plate and SHPB models may suffice. However, to combine biological realism with accurate blast modeling, shock tube experiments may be the best option to investigate bTBI mechanisms in a laboratory setting.

### 4.2. Simulations and Computational Methods

Simulations are among the most cost effective methods for studying bTBI and cavitation, across a range of length and time scales. For example, molecular dynamics (MD) and atomistic simulations are a good choice to study nanoscale bubble collapse, and gather information about direct impacts on cellular components (10, 65, 78). In the molecular dynamics simulations, bubbles with 10, 20, and 40 nm diameters were placed next to a lipid bilayer. The strength of the shock wave is varied by assigning the piston different velocities (1.0, 1.5, and 2 km/s). Over the course of 5 ps, Adhikari et al. (10) found that collapsing nanobubbles could disrupt simulated BBB tight junctions. Using a piston set at 1 ps at 1, 2, and 3 km/s, Wu and Adnan (65) collapsed 8 and 10 nm bubbles with a peak overpressure of 1 GPa. These MD simulations showed that cavitation bubble collapse led to large scale deformation, or even rupture of hyaluronan (a component of the perineuronal net).

Finite element (FE) simulations of blast scenarios can be performed to help avoid the ethical obstacles that present themselves when animals would otherwise have to be subjected to traumatic injury. In FE modeling, blast simulations can provide a much more feasible way to examine different blast scenarios once validated. Some FE models even suggest that inclusion of the whole body, rather than just the head, is critical for bTBI models (79, 80). In these simulations, the body of a male was placed 2.34 m away from a 2.3 kg (5 lbf) Composition C4 explosion. The peak overpressure on the head was 600 kPa with a peak duration of 0.5 ms. Results from (79) showed that maximum principal strain in the full body model was significantly larger when compared with the head-only model, which suggests that a whole body model is better suited at predicting bTBI. Nevertheless, there are many works which model blast exposure using only head models, but a large portion of these do not address or attempt to model cavitation (4, 39, 81). The head models typically consist of the skull-brain system or skull-fluid system. Zhu et al. (39) utilized a 0.305 m diameter shock tube with a driver length of 0.762 m long, and a driven length of 6.1 m in conjunction with Mylar diaphragms to validate results from their FE simulation. The FE simulation used an arbitrary Lagrangian-Eulerian fluid-structure coupling algorithm to model the interaction between the blast wave and head surrogate. Data from the FE simulation by Zhu et al. (39) showed that increasing the elastic and bulk modulus of the shell and core, respectively, led to significant increases (7.2%) in overpressure. The shell represented the skull and the core represented the brain matter. Another FE model found that smaller blast distances correlated with higher overpressures and more severe injury (82). In these simulations, the distances considered ranged from 3 to 5 m, relative to the source force with overpressures that ranged from 98 to 1,000 kPa for durations of 3–5 ms. Injury severity was assessed by linear and rotational acceleration of the head. For instance, the simulation showed that primary blast loading could lead to linear head accelerations over 2,000 $\frac{m}{s^{2}}$. This was for the case when the blast was modeled by 5 kg of C4, at a 4 m standoff distance. The peak rotational accelerations in the saggital plane reached over 400 $\frac{rad}{s^{2}}$, for this case.

Using FE models involve a variety of complex decisions and mathematical relations regarding material properties of biological tissues, equations of state, nucleation, and collapse pressures, among other. There is not a standard set of model decisions and input parameters for use when setting up a FE simulation. Thus, the accuracy of the FE model are a function of the input parameters. Consequently, more experimental data of blast scenarios and the response of the head (or body) to the blast are needed to validate the response predicted by the FE models. In this way, a standardized FE model for bTBI research may be created.

The finite volume method (FVM) has also been utilized in some cavitation research (35, 83). To better model cavitation in water, Brundage (83) added a tensile region to a two-phase Tillotson equation-of-state (EOS). This two-phase Tillotson EOS was compared with alternate EOS models in several FVM simulations. In one such simulation, a spalling experiment was conducted where the pressure was dropped below the cavitation pressure at the spall plane. Vapor was then inserted at the spall plane using the vapor pressure of water. Results showed that the two-phase Tillotson EOS was able to predict the vapor pressure across the spall plane, where existing equations of state could not.

Haniff and Taylor (35) subjected a previously validated head-neck model to a directional 260 kPa air blast, with the assumption that cavitation bubbles have already been formed. These artificially placed bubbles are then oscillated and burst with a 700 kPa compressive wave. While these types of FVM simulations can provide useful insights into how cavitation collapse might affect surrounding tissues or media interfaces, one limitation of such designs is that the models do not capture nucleation. Since the exact cavitation threshold and behavior of CSF remain unknown, data which artificially generates seed bubbles, or injects vapor, cannot be guaranteed to replicate realistic cavitation scenarios.

Modeling cavitation bubble nucleation may provide insight in bTBI mechanisms. However, modeling is further complicated by the fact that the bTBI is directionally sensitive, and local pressure is variable in different cranial spaces (84). These complexities may offer some insight into why there is not more conclusive evidence of cavitation in simulations. For instance, even if the simulations contain all of the components of the head, the anisotropic nature of the brain tissue, local intracranial pressure (ICP) fluctuations, acoustic impedances, and reflection points are not all included within the model and the response has not all be validated experimentally. Thus, preventing a mechanistic understanding of bTBI from being obtained.

Computational models applied in cavitation research are summarized in Table 2.

**Table 2:** A summary of simulations modeling cavitation.

**Computational method**	**Model description**	**Software**	**Pressure ranges**	**Outcome measures**	**References**
Finite element	Computational fluid dynamics simulation of a composition C-4 explosion on a whole body model	CoBi	Up to 500 kPa recorded in brain tissue, duration 1ms	Biomechanical response (displacement, strains, pressure)	(79)
Finite element	Two-dimensional blast wave model with transverse head model	LS-DYNA	50–1,000 kPa pressure waves, 1,500+ recorded in brain, duration ranged 1–8 ms	Biomechanical responses	(36)
Finite element	Three-dimensional head model subjected to oblique and frontal impacts		Up to 750 kPa recorded in brain tissue, duration 6 ms	Biomechanical response	(29)
Reactive molecular dynamics	Nanoscale simulation of bubble collapse in the perineuronal net		System calibrated at 101 kPa, max overpressure 1 MPa, duration not given, bubbles recorded over 10 ps	Structural and biomechancial data	(65)
Finite volume	Microscale model of the white matter with collapsing cavitation bubbles	CTH	Bubble collapse generated up to 700 kPa in the ECM, duration 60 ns	Biomechanical response	(85)
Finite volume	One-dimensional water flyerplate model to test novel EOS	CTH	3,000 kPa in flyerplate impact plane, test duration 45 μs	Pressure, temperature data	(83)

## 5. Approaches and Outcome Measures to Assess Cavitation Damage

Differentiating histopathological and behavioral changes caused by a shock wave from changes caused by cavitation is challenging. Thus, the following subsections discuss the limitations of existing histopathological and biomechanical outcomes in cavitation research.

### 5.1. Histopathology, Biomarkers, and Behavior

Histopathology can thus far only be used to asses artificially generated cavitation (i.e., seed bubbles on hippocampal slices) (12). Currently, there is no published research validating shock wave-induced cavitation in a tissue model. Even if such a model were developed, it is difficult to differentiate specific changes in the tissue model attributed to cavitation from other blast-tissue effects (e.g., spallation, implosion, and inertial forces). However, several researchers have found injury signatures which may be unique to blast impacts, such as distinctive diffuse axonal injury (DAI) patterns in bTBI, which are discussed below. In addition, bTBI seems to lead to unique tissue tears and shearing injuries at the coup and contrecoup sites (which are also likely sites of cavitation) (24). In rats, blast exposure lead to altered gene expression, with a downregulation of genes associated with neurogenesis and synaptic transmission, but did not necessarily display signs of diffuse axonal injury (86). On the other hand, veterans with a history of blast exposure did show amyloid precursor protein positive swellings consistent with diffuse axonal injury (DAI). However, they had a pattern and colocalization with ionized calcium binding adaptor molecule 1 positive [IBA1 (+)] microglia not seen in blunt injury or opioid overdose (67). The idea that bTBI leads to a unique pattern of DAI and vasospasm is supported by additional research (66).

Histopathological data may not be practically useful in the majority of cavitation research, due in part to the obstacles related to cavitation production by a blast wave in an experimental setting. Furthermore, isolating the effects of cavitation from other possible injury mechanisms is another challenge of histopathology. Thus, blast specific injury signatures are a key aspect of bTBI research that need further exploration. Identifying, with more certainty, what the blast-specific hallmarks are and if they consistently exist across blast victims will help identify the mechanisms leading to such injuries.

There has been some attempt to identify biomarkers affiliated specifically with cavitation bubble collapse. Responses to cavitation bubble collapse vary with cell types (e.g., microglia, astrocytes, and neurons), but consistently lead to altered gene expression and mitotic activity (31). Cavitation injury specifically has been shown to be capable of the bast-typical parenchymal lesions and shearing injuries discussed in the preceding paragraph (31). In fact, a cavitation based model was found to reliably produce histopathology identical to other brain injury models (31). Furthermore, cavitation has been shown to increase inflammatory cytokine levels in multiple cases (31, 87). However, these models utilized commercially available extracorporeal shock wave devices. Extracorporeal shock wave exposure is highly distinct from a blast injury scenario in almost all of the determining criteria for the injury (peak overpressure, blast duration, distance from the blast, and degree of reflection). So, while these studies may show that ESWs can lead to comparable injuries to blast wave exposure, their results must be viewed cautiously as supportive or antagonistic to any findings from studies that reflect more accurate blasts.

Similar to histopathology, behavioral data from cavitation is not well-documented. Although studies examining behavioral outcomes in blast trauma experiments exist, they do not evaluate whether or not cavitation was occurring in their models (53, 88). In a study where cavitation was occurring, rats did display typical behavioral and cognitive deficits (31).

In summation, histopathology, biomarkers [e.g., glial fibrillary acidic protein (GFAP), tau protein], and behavioral outcomes of blast traumatic brain injury are still not fully characterized at all, but are also not likely to be helpful in resolving the outstanding questions in cavitation research. For pathological data to be helpful in evaluating the likelihood of CSF cavitation, it would be necessary to separately expose animals to a cavitation injury versus a blast where there was no cavitation occurring. This is almost certainly not realistic, so while pathological and biological characterization of blast injury is key research for the medical side of injury, it is unlikely to be helpful in deciphering the mechanisms behind blast injury.

### 5.2. Biomechanical Measures

Cavitation is a well-studied problem in non-biological settings. Biomechanical outcome measures (e.g., pressure data, deformation, kinematic data) utilized in these settings can be adapted for biological settings. One such biomechanical measure is to record pressure data. Since it is not always possible to visually confirm cavitation, pressure data is often recorded and utilized as suggestive evidence of cavitation, with negative pressures necessary for nucleation, and strong positive pressures often forming on collapse (81, 89, 90). Additionally, intracranial pressure measures can provide information on areas inside the head where cavitation may occur (34, 35). Measurements of incident overpressure (i.e., primary shock wave) can often be correlated with biological measures like ICP, to study bTBI (91). Several other examples of studies which examined pressure data are discussed in section 4.

In addition to pressure data, biomechanical studies of cavitation can provide valuable insights into head kinematics. Blast directionality and peak overpressure are key factors which may affect kinematics (91). For example, Feng et al. (91) found a linear correlation between incident overpressure and linear and angular acceleration of a pig head in a spherical 3.6 kg C4 explosion (overpressures of 150, 300, and 400 kPa). Among others, Singh and Cronin (82) examined head kinematic data attributed to blast exposure, as discussed in section 4.2. The results describing the linear and rotational head acceleration may give insight into the kinematic conditions that would yield cavitation.

Another common biomechanical measure involves deformation of soft materials (e.g., brain tissue, gels, polymers). Deformation and subsequent measures like strain can be obtained using the digital image correlation (DIC) techniques (92). Specifically, DIC calculates the strain on the soft material surface, which can then be used to analyze areas of likely cavitation injury, and provide data to help validate computational bTBI models (36, 85).

Overall, biomechanical measures like intracranial pressure, head and brain kinematics, and deformation, are well-suited to this interdisciplinary research due to their ability to provide translationally useful data for both engineers and biologists. Obtaining some of these measures, like ICP, is often logistically easier than attempting to record cavitation in real time. These biomechanical outcome measures, regardless of ease of acquisition, are all beneficial due to the continued absence of distinct biomarkers or pathological hallmarks of cavitation.

## 6. Discussion and Future Directions

Despite consideration of cavitation as a blast injury mechanism for over 50 years, almost none of the fundamental questions about cavitation have been conclusively answered. First and foremost, there is still no confirmed instance of human cerebrospinal fluid cavitation during a blast wave. Even though, there is an abundance of theoretical and experimental support for cavitation occurring in response to a blast. Since the cavitation threshold of human cerebrospinal fluid has not been characterized, it is unclear if negative pressures from a non-lethal blast is sufficient to enable nucleation, skull flexure, reflection, and local pressure variation within the cranial spaces needed to theoretically create conditions where nucleation could occur. Proving cavitation in CSF has been difficult due to the combination of ethical concerns, logistics, and the transient nature of cavitation.

This brings forth an additional set of questions, however. Even if cavitation occurs during blast trauma, the mechanism through which it leads to injury is even less clearly documented than how cavitation occurs. Existing studies on biological and behavioral hallmarks of bTBI do not usually mention cavitation and are not generally helpful in evaluating the presence of cavitation. However, on the purely mechanical side, there are many considerations about the complexities of the head which need to be taken into account. Variable pressure within the head, tissue-fluid boundaries, the properties of the CSF (volume, flow rate, ionic composition), may all affect the cavitation behaviors of the cerebrospinal fluid.

Reviewing current literature on CSF cavitation in blast injury comes with several limitations. First and foremost, CSF cavitation research is spread across two already complex disciplines, biology, and engineering. The conclusions which can be drawn after attempting to synthesize both fields are somewhat limited by nature, since experiments designed to characterize bubble dynamics do not necessarily account for realistic biological parameters, and vice versa. To obtain conclusive evidence of CSF cavitation as a bTBI mechanism, experiments must take into account realistic blast conditions and structurally complex biological models, and must do so while also being able to record proof of the transient cavitation event. Given these complexities, it becomes less surprising that despite CSF cavitation's presence in the bTBI literature for over 50 years, its existence has not been demonstrated. This lack of evidence potentially limits the conclusions of this review, while also perhaps highlighting its need.

Much of the existing experimental literature evaluating how CSF cavitation could occur, or damage the brain, is based on assumption of its likely occurrence. It is the aim of this review to demonstrate that this widespread assumption is not currently supported by data. This lack of fundamental evidence make drawing conclusions about the likelihood of CSF cavitation difficult. Thus, the position of the authors regarding future blast injury and cavitation research is two-fold. First, is that cavitation research should continue in conjunction and with increased consideration of other possible mechanisms. Continued research on cavitation is the only way to resolve the many unanswered questions underpinning its likelihood as a bTBI mechanism. However, many other possible explanations like inertial effects and skull deformation are under-explored compared with cavitation. Exploring other mechanisms serves as a means of addressing the CSF cavitation question using the process-of-elimination concept, while providing insight into bTBI mechanisms which cavitation research has so far struggled to produce. This is not to suggest that CSF cavitation is not important and relevant for future blast research, but merely that it ought to be studied as one component of many other possibilities that seem equally as feasible. Furthermore, cavitation research should not only focus on the CSF layer. Tissue shearing and parenchymal lesions in blast injury are not restricted to the ventricular lining or tissue-CSF boundary, as one would expect if CSF cavitation was the sole mechanism of injury. CSF cavitation is a valid focus of proof-of-concept research, but is not likely to be solely responsible for blast pathology.

The authors' second position is that the critical next step of CSF cavitation research is one which should have perhaps preceded much of the existing literature. That is, experimentally validating the cavitation threshold of human CSF. Due to ionic composition alone, it is possible that the cavitation threshold of CSF is divergent from that of water. This threshold needs to be determined, if for no other reason than to validate the use of CSF stimulants in prior research. Admittedly, the geometry and flow of CSF within the head, as well as the complex nature of the brain tissues' response to a shock wave, make accurate modeling difficult. However, if a small, non-flowing volume of CSF does not cavitate at non-lethal overpressures, this would be a good indication that widespread CSF cavitation is not a primary mechanism of blast injury. Cerebrospinal fluid cavitation has occupied a prevalent position in bTBI literature for a number of years, despite a general lack of conclusive experimental data. Moving forward, mechanical testing approaches like shock tubes, and finite element models, should be combined with more refined biological data (e.g., complex head models or *post-mortem* heads so as to most accurately reflect geometric intricacies, use of real CSF samples, as opposed to simulants). In this way, the best possible analysis of the likelihood of widespread CSF cavitation can be obtained. Until then, great caution is needed when providing conclusions regarding CSF cavitation in realistic blast scenarios.

Addressing the still remaining question of whether cavitation occurs *in vivo* will require imaging techniques, in real time, of live animals exposed to a blast. Accomplishing this task is both logistically and ethically challenging. To image cavitation during blast exposure, the CSF system must be visible in a living animal to facilitate recordings using high-speed cameras. This could theoretically be accomplished using *Xenopus tropicalis* or another species that have a period of skull/brain transparency. Xenopus have been used to record live CSF flow before (93), so similar techniques could be applied while exposing the animals to shock. However, the generalizability of the results from the *X. tropicalis* to primates and humans will be questionable. Thus, other options to visualize a potential cavitation event could involve optical clearing or fluorescence microscopy. Optical clearing of the skull will create a cranial window, which is beneficial for *in vivo* imaging of the cortex to study problems in neuroscience (94). This method uses a skull optical clearing solution (SOCS), developed by Wang et al. (94), to view the structural and functional organization of the cortex without performing a craniotomy. The use of the SOCS may be a potential method to view a cavitation event. Fluorescence macroscopy has already been used for *in vivo* imaging of CSF transport in rodents (95). The results from the study by Sweeney et al. (95), showed that fluorescent macroscopy could be reliably used to visualize CSF dynamics through the skulls of live mice. The protocols used a cisterna magna cannula to inject commercial tracers into the CSF system. An imaging window was created by shaving the animal's fur and removing the skin. A layer of cyanoacrylate glue can be applied to protect the exposed skull from infection, without interfering with imaging. The authors went on to use transcranial macroscopic fluorescent imaging to study glymphatic transport using commercially available fluorescent tracers. Therefore, it is feasible that fluorescence macroscopy could be adapted to a model where rats are exposed to a survivable shock wave delivered via a shock tube to visually record CSF response and check for cavitation bubble formation.

Another option for cavitation imaging is ultrasound. Ultrasound has been used in prior studies to detect cavitation events as soon as 330 μs after ultrasonic excitation (96). However, there are several reasons that alternative methods like optical clearing or fluorescence macroscopy should be considered instead of ultrasound imaging. First, blast models often use adult animals with fully fused skull bones, limiting the use of ultrasound. Additionally, the axial resolution from ultrasound can be insufficient to identify nucleation sites when imaging for cavitation (97). Previous attempts by Vignon et al. (97) to image cavitation, using ultrasound, noted difficulties with temporal discontinuity between frames and excitation pulses, as well as complex backscatter. Certainly, these challenges are not insurmountable, and ultrasound imaging remains a good potential candidate for monitoring cavitation *in vivo*. However, these documented challenges also highlight the usefulness in exploring alternate imaging strategies.

In summation, drawing conclusions of CSF cavitation as a bTBI mechanism is challenging since the literature is spread across differing disciplines (e.g., biology and engineering). Thus, interdisciplinary collaborations will be invaluable at designing future CSF cavitation experiments to uncover new and critical insights into injury pathways in blast trauma. These interdisciplinary collaborations will also be key when investigating alternative bTBI mechanisms such as spalling, inertia, and implosion, which may occur in conjunction with cavitation. If cavitation continues to be considered as a potential bTBI mechanism, then there first needs to be conclusive evidence that cerebrospinal fluid cavitation can occur at a blast level that is nonlethal to the animal.

## Author Contributions

JM primarily conducted the literature review and drafted most text of the review, as well as generating figures and tables. SB is the PI of the lab, edited and assisted in drafting, and composing the review, figures, and tables. Both authors contributed to the article and approved the submitted version.

## Conflict of Interest

The authors declare that the research was conducted in the absence of any commercial or financial relationships that could be construed as a potential conflict of interest.
